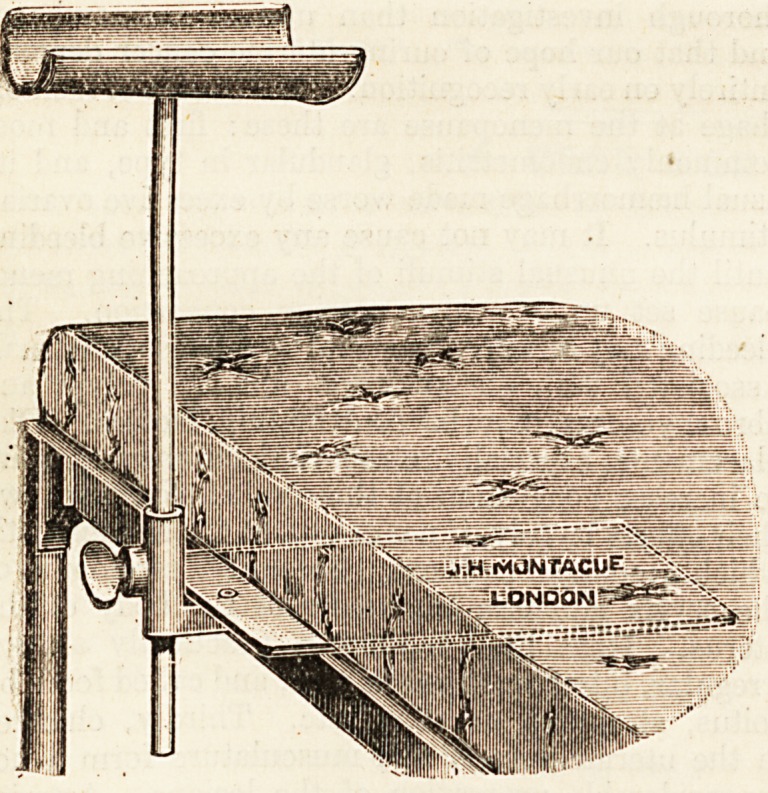# Lumbar Operations on the Kidneys

**Published:** 1909-03-06

**Authors:** 


### ANÆSTHETICS.

### LUMBAR OPERATIONS ON THE KIDNEYS.

For these operations the administrator should be influenced in his choice of an anæsthetic partly by the general state of the patient, partly by the manner in which the respiration and circulation are liable to be affected by the position employed, and by the surgeon's manipulations, and partly by the various complications of which he can foresee the possibility.

There has been much discussion in regard to the effect of ether inhalation on the kidneys (especially when these are disordered) and the consequent advisability of its use in operations on those organs. The balance of opinion appears to be that there is no well proved reason for discarding ether on this account. On other grounds ether is, however, unsuitable for most cases. The lateral position, even when the arm-rest (presently described) is employed, hampers the expansion of the chest; cough must on no account be allowed, as it is annoying to the operator, and may result in such a catastrophe as a wounded or torn blood-vessel, or even a rupture of the peritoneum; consequently accumulation of mucus and cyanosis are apt to occur, with associated rigidity of muscles, venous oozing, and disturbance of the action of the heart. If ether be given on account of feebleness, as in a person anæmic from hæmaturia, anything like intercurrent asphyxia through the too prolonged or close application of an inhaler should be carefully avoided.

As regards chloroform, there is no doubt that reflex disturbances of vital functions are more apt to attain a serious degree when this anæsthetic is employed alone than when ether or a mixture of chloroform and ether is given. Such reflex disturbances are almost always present to some extent during kidney operations; and, therefore, except in robust subjects and for comparatively short operations, it is better to use a mixture (A.C.E. or C.E.).

Careful attention should be given by the anæsthetist to the position of the patient, in which he is as much concerned as the surgeon. A small firm cushion, not an ordinary pillow, should be placed under the head, and it should not project beyond the face, so that room is afforded for the application of the mask and for free exchange of air. The uppermost arm should be well supported at a right angle to the body. The patient can then breathe more freely, and is steadied and less likely to slip into a prone position. A useful arm-rest is that described in the *British Medical Journal*, Dec. 15, 1900 (see illustration). If this be not forthcoming the arm should rest on a box placed on a small table or something similar. The loin cushion if too broad or placed too high will interfere with the respiration.

It will generally be found that there is some difficulty in keeping, with due regard to safety, a level anæsthesia whilst the patient is being adjusted as required by the operator, and his sensibility must be carefully tested before the surgeon makes his incision, otherwise he may plunge out of position.

Manipulation of the kidney, especially when it is drawn out, is frequently accompanied, even under deep anæsthesia, by laryngeal spasm and various disturbances of pulse and respiration. The anæsthetist must maintain a free airway by pushing forward the lower jaw and holding out the tongue, deepening the anæsthesia if this seems insufficient, as judged by other signs. When pressure is made on the kidney by an assistant from the front, some fluid may be forced from the stomach with little or no retching, and the anæsthetist should be on the watch for this. On ligature of the pedicle there may be some signs of shock, even when ether is being given. It is, therefore, very needful for the anæsthetist to keep himself aware of the actions of the operator and his assistants, but he must not allow his attention to be diverted from continuous observation of the patient by too keen an interest in their proceedings.

Anæsthesia must be well maintained until the application of the bandage, especially if an extensive wound has been made with exposure of peritoneum. Premature return to consciousness with straining and vomiting would here lead to much mischief.

Lastly, although it is desirable for certain reasons that the bandage should be put on tightly, the anæsthetist should see that it is not applied in such a way as to impede seriously the respiration.

## Figures and Tables

**Figure f1:**